# Chemotherapy-Induced Hepatotoxicity in HIV Patients

**DOI:** 10.3390/cells10112871

**Published:** 2021-10-25

**Authors:** Silvia Bressan, Alessandra Pierantoni, Saman Sharifi, Sergio Facchini, Vincenzo Quagliarello, Massimiliano Berretta, Monica Montopoli

**Affiliations:** 1 Department of Pharmaceutical and Pharmacological Sciences, University of Padova, 35122 Padova, Italy; silvia.bressan.6@phd.unipd.it (S.B.); alessandra.pierantoni@studenti.unipd.it (A.P.); saman.sharifi@phd.unipd.it (S.S.); monica.montopoli@unipd.it (M.M.); 2 Università della Svizzera Italiana (USI), 6900 Lugano, Switzerland; 3 Veneto Institute of Molecular Medicine, 35129 Padova, Italy; 4 Department of Precision Medicine, Division of Medical Oncology, “Luigi Vanvitelli” University of Campania, 81100 Naples, Italy; sergio.facchini@studenti.unicampania.it; 5 Division of Cardiology, Istituto Nazionale Tumori-IRCSS-Fondazione G. Pascale, 80131 Naples, Italy; v.quagliariello@istitutotumori.na.it; 6 Department of Clinical and Experimental Medicine, University of Messina, 98122 Messina, Italy; 7 Gruppo Oncologico Ricercatori Italiani, GORI ONLUS, 33170 Pordenone, Italy

**Keywords:** drug-drug interactions, hepatotoxicity, HIV, ART, chemotherapy, immunotherapy

## Abstract

Human immunodeficiency virus (HIV) affects more than 37 million people globally, and in 2020, more than 680,000 people died from HIV-related causes. Recently, these numbers have decrease substantially and continue to reduce thanks to the use of antiretroviral therapy (ART), thus making HIV a chronic disease state for those dependent on lifelong use of ART. However, patients with HIV have an increased risk of developing some type of cancer compared to patients without HIV. Therefore, treatment of patients who are diagnosed with both HIV and cancer represents a complicated scenario because of the risk associated with drug–drug interaction (DDIs) and related toxicity. Selection of an alternative chemotherapy or ART or temporarily discontinuation of ART constitute a strategy to manage the risk of DDIs. Temporarily withholding ART is the less desirable clinical plan but risks and benefits must be considered in each scenario. In this review we focus on the hepatotoxicity associated with a simultaneous treatment with ART and chemotherapeutic drugs and mechanisms behind. Moreover, we also discuss the effect on the liver caused by the association of immunotherapeutic drugs, which have recently been used in clinical trials and also in HIV patients.

## 1. Introduction

The assessment of Antiretroviral Therapy (ART) into the clinical setting has had a significant impact on the clinical outcomes of HIV-related cancers. The range of cancers diagnosed among people living with HIV (PLWH) includes so-called AIDS-defining cancers (ADC—Kaposi’s sarcoma, invasive cervical carcinoma and non-Hodgkin’s lymphoma) and non-AIDS-defining cancers (NADC—Hodgkin’s disease, anal, lung, breast, skin, colorectal cancer, hepatocellular carcinoma, etc.) [1,2]. Due to the introduction of ART, a decrease in ADCs and an increase in NADCs were observed due to the aging of PLWH cancer patients. The challenge in the treatment of HIV-related cancers is the need to maintain an adequate management of HIV infection during the antiblastic chemotherapy (AC), target therapy (TT) and immunotherapy (IT), reduce the risk of opportunistic infections (OIs) and finally to restore immunomodulator activity.

It is clear that patients who receive a combination of AC, TT, IT and ART can achieve better response and survival rates than patients who receive AC alone. The combined treatment between ART and AC, TT and IT is feasible and reduces the incidence of OIs. However, careful attention must be paid to cross toxicity and possible pharmacokinetic and pharmacodynamic interactions between ART and AC, TT and IT. Drug–drug interactions (DDIs) occur when one drug influences the level or activity of another when concurrently administered. They may result in increased therapeutic or adverse events, decreased therapeutic or toxicity or a single response that does not occur when either agent is administered alone.

DDIs arise at all levels and a failure in their identification is responsible for patients’ overdosing or under-dosing. DDIs represent a primary concern in treatment, and they are usually more prevalent in a cancer setting. This could be due to the narrow therapeutic index and the inherent toxicity of AC. The risk of DDIs has been found to increase with the number of simultaneous medications [3]. Cancer patients receive many drugs during their therapies including those for comorbidity and cancer-related symptoms such as pain, depression, and emesis. Therefore, they are at increased risk to develop DDIs. According to Corona and colleagues, DDIs are frequent in oncology [4]. In most cases, the consequences of DDIs are unwanted, compromising the effectiveness of the therapeutic agents or enhancing their toxicity [5]. It has been reported that about 20–30% of all adverse drug events are caused by interactions between medications. To date few partial data are available on DDIs in the treatment of HIV-associated cancers, especially in TT and IT settings. Protease inhibitors (PIs) and non-nucleoside reverse transcriptase inhibitors (NNRTIs) are potent inhibitors/inducers of the cytochrome P450 (CYP450) metabolic system. Since many AC are also metabolized by the CYP450 system, co-administration with ART could result in drug piling up and possible adverse event or decrease the efficacy of one or both types of drugs [6].

Amplified toxicity may lead to a delay of AC, TT and IT recycling or to a prompt dose reduction, possibly compromising the therapeutic benefit of these treatments [7]. Toxicity can also negatively affect ART compliance, favoring the emergence of resistant HIV strains. Recent data have shown that toxicity, particularly myelosuppression, and neurotoxicity, is significantly more common in patients treated with combined therapy than in patients treated with AC alone [8]. Alternatively, patients treated with AC and ART have a better survival rate than patients treated with only AC, suggesting that the reduction of OIs complications due to ART with the consequent amelioration of their performance status (PS), can improve the overall outcome in the combined treatment setting [9,10]. This paper focuses on the risk of potential interactions and subsequent therapeutic considerations in the combination of ART and AC, TT and IT used in the treatment of PLWH cancer patients. Unfortunately, few data are available about the risk of interaction between, IT and ART due to recent advent of this kind of treatment in cancer patients.

## 2. ART and Drug Metabolism

### 2.1. Nucleoside or Nucleotide Reverse Transcriptase Inhibitors (NRTIs)

HIV reverse transcriptase (RT) is an RNA-dependent DNA polymerase, which converts viral RNA into DNA before its incorporation into the host cell DNA by viral integrase. The inhibition of this enzyme is possible with the administration of either nucleoside or nucleotide reverse transcriptase inhibitors (NRTIs) or nonnucleoside RT inhibitors (NNRTIs) [11]. Reverse transcriptase inhibitors are mainly categorized as nucleoside analogues, apart from tenofovir, which is a nucleotide analogue. NRTIs can be formulated as prodrugs or drugs. When NRTIs are administered as prodrug, they are first metabolized by the cell to their drug form; and subsequently phosphorylated to its active form, diphosphate (DP) or triphosphate (TP). When NRTIs are activated, they act like functional nucleoside analogues, able to block the enzymatic function of HIV-1 RT, and as a result, viral DNA cannot be incorporated into the host cell DNA. This class of drugs is not an inhibitor for the Cytochromes P450 enzymes; therefore, possible DDIs are unlikely. However, NRTIs class can lead to mitochondrial toxicity, indeed they can also block mitochondrial DNA (mtDNA) polymerase due to its similarity to the HIV-1 RT. The result is the production of dysfunctional mitochondrial protein; the accumulation of these proteins results in mitochondrial toxicity that can induce myopathy, neuropathy, lipoatrophy, and lactic acidosis, with or without hepatic steatosis [12].

### 2.2. Non-Nucleoside Reverse Transcriptase Inhibitors

Non-nucleoside reverse transcriptase inhibitors (NNRTIs) are essential components in ART due to their unique antiviral activity, high specificity, and low toxicity. NNRTIs inhibit HIV-1 RT by binding and inducing the formation of a hydrophobic pocket located around 10 Å from the catalytic site of the p66 subunit of the enzyme; however, efficacy for the NNRTI drug class is reduced by mutations within or near the NNRTI binding pocket [13]. Over 50 diverse chemical groups have been identified as NNRTIs targeting RT, notwithstanding that the US FDA has approved nevirapine (NVP), delavirdine (DLV), efavirenz (EFV), etravirine (ETR), rilpivirine (RPV), doravirine (DOR) and Russian Ministry of Health (MoH) has also recently approved elsulfavirine [14]. Each NNRTI interacts with different amino acid residues in the NNRTI binding pocket (NNIBP); nevertheless, they can be substrate or inhibitor of Cytochromes P450 (CYP) enzymes. Indeed, EFV is a substrate of CYP2B6, but also a week inhibitor or inducer of CYP2A4. ETR, which is a second-generation NNRTI, is mainly used in patients who have developed resistance against other antiretroviral drugs. It is a substrate for CYP4A; however, it can be also a weak inhibitor of CYP2C9 and CYP2C19. The inhibition of CYP can lead to drug–drug interactions (DDIs), increasing drug exposure and possible toxicity. Since a lot of drugs used for both treating possible OIs or AC are a substrate for CYP, the possible inhibition of the latter could lead to a toxic drug accumulation. 

### 2.3. Protease Inhibitors (PIs) 

HIV-1 protease is responsible for the production of viral enzymes and structural proteins, essential for the assembly of virulent virions. Therefore, the inhibition of HIV-1 protease with Protease Inhibitors (PIs) introduce a block at a vital stage in the HIV life cycle [15]. One of the main problems with PIs is that HIV-1 protease is prone to mutation, leading to the development of PI resistance. These mutations can be classified into two groups: primary and secondary mutations. The first is characterized by the changing of residues directly involved in the substrate binding and results in alteration in the interaction between the protease and PI. The secondary mutations are localized in the flap region of the enzyme, consequently leading to a change in the shape of the binding pocket, reducing the ability of PIs to bind to the active site. Hence, drug resistance is one of the possible problems when PIs treatment is administrated [16]. Furthermore, all the PIs are metabolized via the CYP pathway; besides, most of them are substrates and inhibitors of CYP3A4; therefore, DDIs are also possible, not only with AC but with statins, antituberculosis, antifungal and anticonvulsants drugs. Indeed, monitoring and dose adjustment is needed when these drugs are administered in a PIs regiment [17]. 

### 2.4. Integrase Strand Transfer Inhibitors (INSTIs)

The integrase (IN) enzyme catalyzes insertion of the viral DNA (vDNA) into the host’s genome. Integrase strand transfer inhibitors (INSTIs) inhibit HIV by blocking the strand transfer step of viral DNA integration into the host genome. The INSTI class of antiretrovirals contributes to the enhanced safety and efficacy in modern ART regimens; indeed, they are employed in combination with other antiretrovirals classes and in the case of ART, associated drug resistance. Moreover, most of them are not involved in the CYP pathway, and they are not inducers or inhibitors of this class of enzymes. Therefore, possible DDIs are more unlikely to happen, especially with AC. The exception is elvitegravir, which is an inducer of CYP2C9, and must be co-administered with cobicistat, a potent CYP3A4 inhibitor. Any drug that is a strong inducer or inhibitor of CYP3A and/or UGT1A1 may influence the plasma concentrations of INSTIs. Therefore, elvitegravir/cobicistat is commonly associated with metabolism-related drug–drug interactions. INSTIs are generally associated with lower rates of adverse effects than other antiretroviral classes; however, one of the main side effects is a weight gain in a INSTIs regimen [18]. 

### 2.5. CCR5 Receptor Antagonists

C-C chemokine receptor 5(CCR5) is a cell membrane protein from the G protein-coupled receptors (GPCR) family. CCR5 is implicated in the HIV entrance method; indeed, it is a co-receptor that HIV-1 uses to bind cells before viral fusion and entry. It is possible to inhibit the biding between HIV and the CCR5 of the host cell by using CCR5 Receptor Antagonists. Maraviroc is the first oral inhibitor approved by the FDA, and it inhibits the binding of HIV to host cells by competitively and selectively binding to CCR5. It is a substrate for the CYP3A4 cytochrome; however, it is neither an inducer nor inhibitor of any of CYP enzymes. Therefore, possible DDIs, especially with AC, are unlikely to occur [19].

### 2.6. Fusion Inhibitors

HIV-1 infection begins with the attachment of the virion to a host cell by its envelope glycoprotein, which subsequently induces fusion of viral and cell membranes to allow viral entry. The very first steps of the infectious cycle of HIV are attachment, fusion and entry of viral particles in the human cells. During this phase, HIV glycoproteins such as gp120, and gp41, play a crucial role. The envelope protein gp120 binds the CD4 receptor on the host cell surface, starting a cascade of conformational changes in gp120 that exposes the chemokine receptor binding domains and allows them to interact with the target receptor [20]. The main co-receptors used by HIV-1 for entry into the cell are the chemokine receptors CCR5 and CXCR4. Enfuvirtide binds to gp41, preventing the formation of an entry pore for the capsid of the virus, keeping it out of the cell. Since Enfuvirtide is not metabolized and it is not an inhibitor or an inducer of CYP, possible DDIs are unlikely.

## 3. AIDS Defining and Non-Defining Cancers

**Figure 1:**
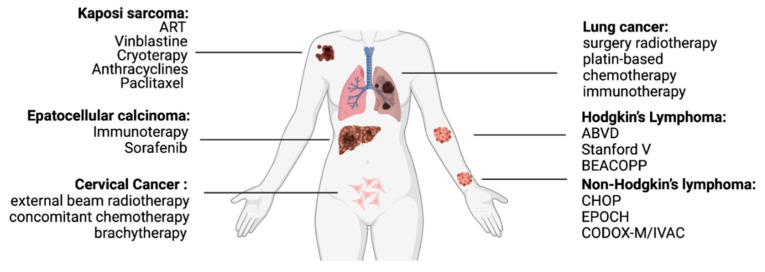
Most common type of tumor in HIV patients.

Besides the incidence of AIDS-defining cancers (ADCs), HIV patients have been also linked to a higher risk of other malignancies, including lung, anus, colon, skin (skin hepatocellular carcinoma), Hodgkin’s disease, and hepatocellular carcinoma. Moreover, if in the ART era the incidence of AIDS-defining cancers has been reduced by the advent of these highly active retroviral drugs, NADCs, on the contrary, have gradually emerged, where HIV-positive patients are estimated to be at 25 times greater risk of receiving a diagnosis of anal cancer, five times greater risk of liver cancer, and three times greater risk of lung cancer when compared with the population without HIV [21]. At present, malignancies represent around 10–20% of all deaths in HIV-positive patients [22,23]. Many reports have noted that the age at cancer diagnosis is 10–20 years younger among people with HIV compared with the general population. Statistically significant is also the difference between the type of cancer, where the youngest population is mostly affected by cancer like lung and anal cancer, while at older ages the diagnosis of Hodgkin lymphoma has been more reported [24,25]. The more aggressive features and poorer outcomes in the HIV population when compared with HIV negative patients is reported in [26] (Figure 1).

### 3.1. Kaposi Sarcoma

Kaposi sarcoma (KS) is one of the most frequent AIDS-related cancers. The viral coinfection of HIV-1 and Kaposi sarcoma-associated herpesvirus (KSHV) [27] is necessary but not sufficient for KS development [28,29,30]. Indeed, it is the immunosuppressing condition due to the HIV-1 infection that has a main role in the KS development. The clinical presentation of HIV-KS is variable; frequently it is presented as cutaneous lesions with a CD4 cells count ≥200 cells/mL and no opportunistic infection ongoing, and it is defined as stage T0. However, it can become an invasive disease with the involvement of deep tissue and/or visceral organs such as lungs, gastrointestinal tract, liver, and spleen, with CD4 cells count <200 cells/mL and possible opportunistic infection occurring; in this case, it is defined as stage T1 [31]. Treatments depend on the stage of disease, symptoms and extracutaneous KS, the HIV viral load, the CD4 count, and the patient’s overall medical condition [32,33]. They can be categorized as topical therapies, physical agents, intralesional chemotherapy, and systemic treatments [34]. As a first line for KS treatment, ART should be started in all diagnoses of KS. In a stage T0 KS, ART alone with PIs could have a significant role in reducing the occurrence of KS [35]. Z Liu et al. showed that patients who had received ART for more than 6 months had a lower incidence of KS compared with people who had received ART for less than 6 months [31], demonstrating the crucial role of this therapy as a valid first-line treatment. However, ART plus the addition of local treatment is always recommended; the canonical regimens are intralesional vinblastine which reported response rates are of 70% [36], oral eposide, and cryotherapy. In a case report, a patient with KS severe foot lesions, that was not responding to the doxorubicin treatment, was treated for six months with intralesional bleomycin, with a complete resolution and regain of foot function. This suggests that bleomycin could also be considered a routine therapeutic option for cutaneous KS [37]. 

For advanced stage of AIDS-associated KS, which is more frequent in the low-income and middle-income countries, the addition of antiblastic chemotherapy (AC) is required. As the first line of chemotherapy pegylated liposomal anthracyclines is highly effective in inducing regression of KS (doxorubicin 20 mg/m^2^ i.v. every 2 weeks or daunorubicin citrate liposome 40 mg/m^2^ i.v. every 2 weeks) [5,38]. Paclitaxel has also been showing to be effective on KS (100 mg/m^2^ i.v.). However, paclitaxel is associated with more hematologic toxicity, more alopecia, and sensory neuropathy concerning pegylated liposomal anthracyclines [39], suggesting that doxorubicin should be considered as the standard treatment. 

### 3.2. Non-Hodgkin’s Lymphoma

Non-Hodgkin lymphoma (NHL) occurs frequently in HIV-positive patients. Before the introduction of ART, the risk of developing NHL in an HIV-positive patient was up to 40-fold more frequent compared to the general population [40]. The introduction of ART has reduced morbidity and mortality from HIV infection and has decreased the risk of developing NHL. However, the incidence of NHL in HIV-positive patients remains elevated compared to the general population. Almost all NHL are of B-cell origin and their development is related to the age of the patient, the CD4 cell count (<100/mm^3^), B-cell dysregulation secondary to HIV infection, the elevated serum lactate dehydrogenase, and no previous treatment with ART [41,42]. Immunosuppression and coinfection with viruses carrying oncogenic proteins, like human herpesvirus type-8 (HHV8) and Epstein-Barr virus (EBV), contribute to HIV lymphomagenesis [43]. The most common NHL subtypes are the large B-cell lymphoma (DLBCL) and Burkitt’s lymphoma (BL). The first-line AC regimens for DLBCL are Cyclophosphamide, Doxorubicin, Vincristine, and Prednisolone (CHOP) regimen with complete remission in 47%. CHOP combine with the monoclonal anti-CD20 rituximab has a complete remission in 56% of cases [44]. The R-CHOP treatment results in increasing the survival of 10–15% without increasing the toxicity. Another combination in this category is Etoposide, Prednisone, Vincristine, Cyclophosphamide, and Doxorubicin (EPOCH) plus rituximab if CD20+. In Burkitt’s lymphoma, the first line of treatment is a combination of cyclophosphamide, vincristine, doxorubicin, and methotrexate/ifosfamide, etoposide, and cytarabine (CODOX-M/IVAC); BL patients treated with this regimen have complete response rates (CRRs) of 75–85%, with almost 65% of patients cured of their disease [45]. 

### 3.3. Cervical Cancer

Cervical Cancer is the most frequently detected cancer in women living with HIV and is classified as an AIDS-defining illness. PLWH have an increased risk of contract cervical cancer compared to women without HIV [46]. Six percent of worldwide HIV-positive woman have cervical cancer but only 5% of all these cases can be linked to HIV [47]. However, in low-income and middle-income countries, the incidence of HIV-related cervical cancer is higher. This is maybe caused by a persistent infection with HPV, which leads to premalignant cervical intraepithelial neoplasia [48,49]. In these cases, HPV vaccination and cervical cancer screening for women living with HIV are extremely important [50]. The standard treatment for locally cervical cancer is external beam radiotherapy, concomitant AC, and brachytherapy. In the case of more advanced disease, the treatment is external beam radiotherapy to the whole pelvis, concomitant chemotherapy plus cisplatin (CDDP) in combination with ART [7]. However, possible interactions between ART and high-risk human papillomavirus (HPV) and cervical lesions in women living with HIV are poorly understood. Studies from Africa and Europe or North America indicate that ART was associated with a lower prevalence of high-risk HPV and cervical lesions. Moreover, ART can prevent cervical lesion incidence and progression, promote regression, and prevent the incidence of invasive cervical cancer. These findings highlight the importance of early ART initiation (before reaching a low CD4 cell count) and sustained effectiveness, in controlling HPV infection and cervical disease progression [51]. 

### 3.4. Hodgkin’s Lymphoma

The incidence of Hodgkin lymphoma (HL) among people living with HIV (PLWH) is 8 to 10-fold times higher than in the general population. HL is a frequent AIDS non-defining cancer; the probability of its occurrence can increase with moderate immunosuppression or with a high CD4 cell count [52]. The exact mechanism of HL incidence in PLWH is not fully understood. However, there are three presumable hypotheses for explaining its appearance: the first one says that ART therapy itself can increase the CD4 cell count, creating a suitable microenvironment for the proliferation of Reed-Sternberg (HRS) cells, hallmark cells of HL. The second hypothesis is that through pro-inflammatory signals HRS cells can activate and attract CD4 cells. Once activated CD4 can stimulate in succession HRS cells. The last theory employs that PLWH with low levels of CD4 cells are more likely to experience serious AIDS-defining events, therefore, creating a competitor risk with HL [53].

Survival in PLWH suffering from HL has greatly increased in recent years, due to the standard ABVD (doxorubicin, bleomycin, vinblastine, dacarbazine) AC regimen. Nevertheless, Stanford V (mechlorethamine, doxorubicin, vinblastine, vincristine, bleomycin, etoposide, prednisone) can also be employed, it is a 12-week therapy regimen with adjuvant radiotherapy. Intensive BEACOPP (bleomycin, etoposide, doxorubicin, cyclophosphamide, vincristine, procarbazine, prednisone) regimen used concomitantly with ART was administered in a small German patients’ group. The treatment was well tolerated with a CR rate of 100% and a 2-year OS of 83%. 

Xicoy et al. showed that the standard ABVD regimen in aggressive forms of HIV-HL provided results comparable to those of patients included in the BEACOPP, Stanford V, and VBEP regimens in terms of CR. Therefore, due to less adverse interaction ABVD should be considered as standard treatment for HL in PLWH.

### 3.5. Lung Cancer

Lung cancer, both small and non-small cell, is the second most common type of cancer both in men and women, with about 235,760 new cases and 131,880 deaths in 2021 only in the United States. About 90% of lung cancer cases are caused by smoking and tobacco; however, there are also other triggering factors like air pollution exposure and chronic infections. Lung cancer is divided into two broad histologic classes, which grow and spread differently: small-cell lung cancer (SCLC) and non-small cell lung cancer (NSCLC). Non-small cell lung cancer (NSCLC) has emerged as the leading cause of cancer-related death in HIV patients. The development of lung cancer in PLWH has been associated with various factors, including exposure to smoking, CD4 count, viral load, duration of immune deficiency, and inflammatory process. The effects of HIV infection on lung cancer treatment tolerability, toxicity and effectiveness are not well known. The standard regiments for LC treatment are surgery, radiotherapy platin-based chemotherapy and immunotherapy. In details Platinum-based regimens have no specific interaction with ART, but cisplatin-induced nephrotoxicity may need dosage adjustment for some antiretroviral drugs, in particular for tenofovir [54]. Surgical resection is the standard of care for early-stage lung cancer; however, for the treatment of PLWH there are conflicting data about this topic. A comparative cohort study evaluating LC surgical outcomes demonstrate that HIV infected patients may continue to experience more frequent surgical complications than uninfected patients [55]. Nevertheless, Sigel et al. demonstrated that surgical complication and 30-day mortality rate in PLWH and uninfected patients are similar. These findings suggest that concerns regarding short-term surgical outcomes associated with the presence of HIV infection should not play a role in treatment decision-making [56]. 

### 3.6. Hepatocellular Carcinoma

Hepatocellular carcinoma (HCC) is the most common type of liver cancer, and it arises from hepatocytes. Worldwide, HCC is the third type of cancer responsible for cancer-related death. 8% of liver cancer cases are secondary to chronic Hepatitis B or C infections. Another risk factor is cirrhosis, a chronic disease where scar tissue replaces liver cells [57,58,59]. 

HCC is becoming an important cause of mortality in patients with HIV, attributed to coinfection with hepatitis C virus, hepatitis B virus, and the longer survival advantage these patients are achieving after introducing the highly active antiretroviral therapy regimens [60,61,62].

In addition to hepatitis infection, immunosuppression secondary to HIV infection, the direct impact of the virus on liver parenchyma, and the use of hepatotoxic antiretroviral drugs, all contribute to HCC pathogenesis [63].

## 4. DDIs between Antiblastic Chemotherapy and ART

Progresses in anti-retroviral therapy led to an increase in the number of people living with HIV worldwide. However, the increment in the overall survival of HIV patients implicate a raise in the amount of PLWH developing AIDS-defining and non-defining malignancies. Use of chemotherapy in concomitance with anti-retroviral therapy is a difficult choice because of the development of DDIs between chemotherapy and ART. Hence, a stable ART regimen can be modified before chemotherapy to reduce toxicity, improve adherence and tolerability, and avoid DDIs. Before starting chemotherapy together with ART, many factors must be considered, such as the degree of immune suppression, the presence of overlapping toxicities and the benefits of HIV viral suppression for the tumor response and survival. When overlapping toxicities are predicted, solutions included the change in the ART therapy, the reduction in the chemotherapeutic dose or the use of an alternative chemotherapeutic strategy should be considered. Besides toxicities, some chemotherapy agents can also interfere with the efficacy of anti-retroviral therapy, mainly by reducing their concentration (Table 1).

### 4.1. Taxanes

Lots of trials have established the efficacy of paclitaxel for the treatment of HIV-KS, with a response rate from 57–66%. The combined treatment of ART and AC is well tolerated. However, paclitaxel is mainly metabolized by CYP2C8, CYP3A4, and CYP3A5. Therefore, a co-administration of paclitaxel and CYP3A4 inhibitors, such as ritonavir, could lead to an increased plasma concentration of the drug. This can develop adverse events such as myelosuppression, liver function test elevations, neutropenia, and peripheral neuropathy. Nevertheless, there are good results with the minor adverse event in a clinical trial with the administration of paclitaxel in combination with CYP3A4 inducers such as efavirenz (NNRTIs) and tenofovir disoproxil fumarate (NRTIs). Paclitaxel was administered as a 1-h infusion at a dose of 100 mg/m^2^ of body surface area following administration of a standard premedication regimen, which contained dexamethasone with H1-receptor and H2-receptor antagonists. Concurrent with AC, all participants received efavirenz (600 mg), tenofovir disoproxil fumarate (300 mg), and emtricitabine (200 mg) [64]. All the patients showed an optimal response to the drug combination with a comparable adverse event to the standard treatment.

### 4.2. The Vinca Alkaloids

The vinca alkaloids are an important class of anti-cancer drugs that are used to treat a wide spectrum of neoplasms. They are administered to treat lung, breast, onco-hematological disease and testicular cancer, and they are also administered for AIDS-related KS. In particular, vinblastine is metabolized by hepatic P450 cytochrome isoenzyme CYP3A4 and it is a substrate for both MRP1 (ABCC1) and P-gp efflux pump in the proximal renal tubule. CYP3A4 is involved in the detoxification of vinblastine, therefore, a concomitant administration with HIV protease inhibitors, which are strong CYP3A4 and P-gp inhibitors, may increase the plasma concentration of vinblastine leading to possible hematological and neurological side effects. In particular, the co-administration of ritonavir, which is an important CYP3A4 and P-gp inhibitor, or Lopinavir that is also a P-gp inhibitor, could increase vinblastine exposure. Vinblastine is also a substrate and inhibitor of multidrug resistance-associated protein 2 (MRP2) transporter in the proximal renal tubule. Inhibition of this renal transporter could increase renal toxicity. 

### 4.3. Etoposide

Etoposide is mainly used in combination with different AC to treat hematological malignancies and NHL. Its metabolism is mediated by CYP3A4 with a minor contribution of CYP1A2A and CYP2E1 isoforms. Thus, the inhibition of the CYP3A4 pathway may increase etoposide plasma concentration levels. Leading to an increased risk of mucositis, myelosuppression, and transaminitis. 

### 4.4. Corticosteroids

Corticosteroids are part of combination AC regimens, and antiretroviral could modulate their biotransformation, leading to changes in their pharmacokinetic and pharmacodynamic. Dexamethasone and methylprednisolone are metabolized by CYP3A4, so a co-administration with ART therapy could lead to DDIs. Prednisone is a substrate for the CYP450 enzyme system, including CYP3A4. This could lead to an increase in toxicity with CYP3A4 and a decreased efficacy with CYP3A4 inducers. Both PIs and NNRTIs are modulators of the activity of the CYP450 enzyme system and therefore may interact with corticosteroids. PIs may increase the pharmacodynamic effects of corticosteroids when used concurrently. Conversely, CYP3A4 inducers may reduce the efficacy of these drugs.

### 4.5. Alkylating Agents (Cyclophosphamide, Ifosfamide)

Cyclophosphamide is used to treat HL and NHL; its metabolism is characterized by two different pathways. CYP2B6 isoenzyme mediates the hydroxylation of Cyclophosphamide, whereas the N-dechloroethylation by CYP3A4 leads to inactive dechloroethyl-cyclophosphamide and chloroacetaldehyde that are associated with neurotoxicity and urotoxicity. Therefore, induction of CYP3A4 may increase neurotoxicity by increasing the substrate availability for N-dechloroethylation, whereas its inhibition should decrease the generation of chloroacetaldehyde minimizing adverse events. Fortunately, only 10% of the administered drug dose undergoes the CYP3A4 pathway. 

Ifosfamide is administered as a racemic mixture of two enantiomers: R-ifosfamide and S-ifosfamide. Its bioactivation in the liver is catalyzed mainly by CYP3A4 and also CYP2B64. CYP3A4 is involved in the generation of both the active moiety and toxic metabolite, so its inhibition could compromise its antitumor activity. Furthermore, CYP3A4 induction can increase the presence of toxic metabolites.

### 4.6. Cisplatin

Cisplatin is used to treat principally advanced cervical cancer, lung cancer and breast cancer; however, there is not a lot of knowledge about DDIs between cisplatin and ART therapy. Cisplatin induces CYP3A4; however, notwithstanding, it is not known if the combination with PIs could have an impact on toxicity and possible adverse events [65].

### 4.7. Anthracyclines (Doxorubicin and Daunorubicin)

Anthracyclines are agents used for treatment of HIV-NHL and KS. Possible DIIs appear to be minimal in concomitance of ART administration. Indeed, doxorubicin is metabolized by the generation of inactive 13 hydroxy metabolite doxorubicinol via the action of the ubiquitous cellular aldoketoreductase enzyme. Therefore, interaction between ART via CYP system appear to be unlikely.

## 5. Immunotherapy in HIV Patients

**Figure 2:**
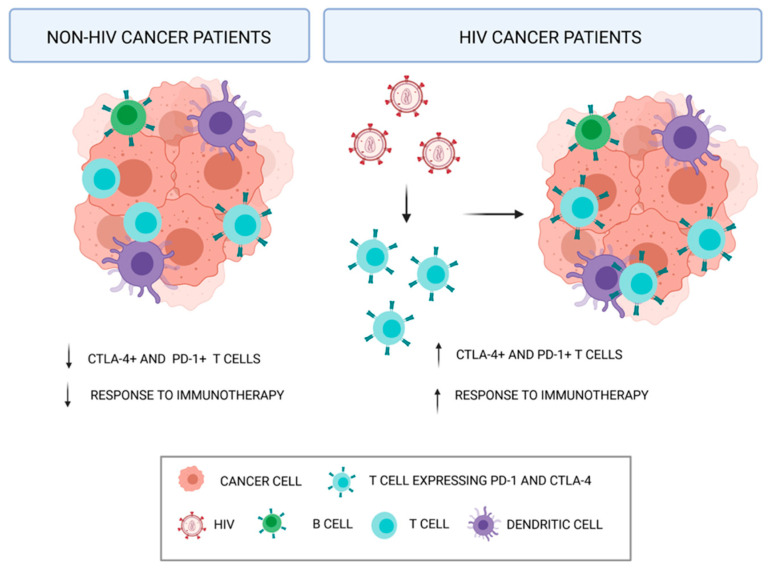
Immunotherapy has been proved to be more efficient in HIV cancer patients compared to non-HIV cancer patients. The increase immune-response is due to the HIV infection itself, which is responsible for the upregulation of the checkpoint inhibitors PD-1 and CTLA-4, as a strategy to suppress the host immune defenses.

Since immunotherapy has been proposed as a novel strategy to defeat cancer, patients with chronic viral infection (such as HIV or hepatitis B or C) have been excluded from pivotal studies because of the decreased functions of cellular and humoral immunity of these patients. However, recent clinical reports show that PLWH present a higher percentage of PD-L1 expressing cells (80%) compared to the population without HIV (30%), giving way to the possibility to apply anti-PD-L1 therapy. Hence, Ostios–Garcia et al. reported that the efficacy and tolerability of nivolumab or pembrolizumab in this small population seems to be at least like that observed in patients who do not have HIV [66]. Application of immune-based therapy maintains an appropriate adherence to ART and both CD4 count, and viral load remain controlled during therapy (Figure 2). 

Moreover, HIV infection has already been proved to be responsible for the upregulation of PD-1 in CD8 cells [67], so the use in HIV positive cancer patients of checkpoint inhibitors affecting the PD-1/PD-L1 axes is a double-edged weapon targeting the infectious disease and cancer. As an example, treatment of lung cancer has been improved thanks to the application of immunotherapy, which mainly acts by interfering in the PD-1/PD-L1 axes, such as nivolumab and pembrolizumab [22]. Indeed, several ongoing trials are being conducted to evaluate anti–PD-1/PD-L1 drugs in patients with advanced NSCLC and HIV infection. The Durst trial is a phase II exploratory study of durvalumab in patients with HIV-1 and advanced solid tumors, including NSCLC. The CheckMate 817 trial is a phase Illb/IVtrial to evaluate the safety and efficacy of nivolumab in combination with ipilimumab, in patients with metastatic NSCLC and it also includes special cohorts such as an HIV-positive population. The IFCT-CHIVA2 trial is a French pilot phase II trial of nivolumab after prior standard AC in PLWH with advanced NSCLC. Pembrolizumab has also been evaluated in a phase I study focused on patients with HIV infection and advanced malignant disease.

Besides, over the past decade, the applicability of immunotherapy in cancer treatment was remarkable in the contest of malignant melanoma, especially for immune checkpoint inhibitors (ICIs). Malignant melanoma incidence in PLWH is increasing in parallel with one of the general populations without differences in terms of incidence and risk rates. Among the HIV-positive subset, no difference is observed among people in the pre-ART and post-ART era. Targeting of blocking programmed cell death protein (PD-1) and cytotoxic t-lymphocytes antigen 4 (CTLA-4) have prolonged malignant melanoma relapse-free, distant metastasis-free, and overall survival times [68].

Surprisingly, HIV infection is responsible for the upregulation of the checkpoint inhibitors PD-1 and CTLA-4 itself, as a strategy to suppress the host immune defenses. Hence, the use of anti-PD1 therapies (nivolumab and pembrolizumab) and anti-CTLA4 (ipilimumab) looks promising for the treatment of malignant melanoma in PLWH. However, until recently, immunotherapies have been not investigated in HIV and most of the available data come from in-vitro, ex-vivo, and in-vivo models. ICIs safety concerns are currently undergoing Phase I trials for PLWH, and all the studies confirm no DDIs [69,70,71].

A case study on the effect of the use of ipilimumab in an PLWH with metastatic melanoma underlines the increased number of CD4+ cells after every injection and an increase in the HIV RNA transcription that likely represents a direct consequence of the anti-CTLA-4 treatment. Indeed, the blocking of the inhibitory effects of CTLA-4 on T cell transcription also translates into a higher viral transcription [72].

Besides classical AC, recently immunotherapy has proven to be effective in classical Hodgkin lymphoma (HL) since frequently genetic mutations lead to the overexpression of the programmed death-1 (PD-1) ligands. However, applicability on PLWH with HL was not assessed in clinical trials, as it happened with other types of cancer since during the first clinical trial in 2015 HIV patients were not involved and in the second one, the HIV status was not reported [73]. Also, in the case of Hodgkin lymphoma, the main checkpoint inhibitors that have been studied are related to the PD-1/PD-L1 and CTLA4 targeting strategy.

The main concern about the application of immunotherapy in PLWH is about the Immune virological evolution, and in 2017 Le Garff et al. reported a case report on the use of nivolumab in an HIV patient with lung cancer and its effect on the immune virological evolution [74]. What they observed was an increase in the CD4+ and CD8+ cells count, higher IL6 blood level, and a decreased number of PD-1 expressing T cells. Interestingly, the patient also developed a Grade I hepatotoxicity after seven nivolumab injections. Initial reports about hepatotoxicity due to immune checkpoint inhibitors show that about 2–30% of patients undergo it, risk of hepatotoxicity increases when using multiple ICIs and in patients who develop other immune-related adverse events. Other risk factors for hepatotoxicity include the underlying chronic liver disease, higher doses of immune checkpoint inhibitors, and utilizing anti-CTLA-4 agents as opposed to anti-PD-1 or anti-PD-LI agents. As regarding toxicity in patients with HIV, still there is a lack of data and information since they are excluded from clinical trials. However, in a prospective study of 2017 publishes in Annals of Oncology, 270 PLWHs patients undergoing an and PD-1 immune checkpoint inhibition for the treatment of NSCLC in combination with ART therapy, were studied and followed up for CD4 count, HIV viral load, and toxicity. Among them, only a patient with Grade 1 hepatitis was detected.

## 6. Hepatotoxicity

A combination of AC and ART present many side effects especially related to renal failure and nephrotoxicity [75]. As previously described, protease inhibitors and chemotherapeutic drugs, such as anthracyclines, vinca alkaloids, taxanes, cyclophosphamide, and etoposides, respectively show high affinity and are metabolized by the same hepatic cytochrome P450 3A enzyme family, and this may affect the toxicity as well as the efficacy of both classes of drugs. Moreover, an interaction of the protease inhibitors with the multidrug transporter P-glycoprotein (P-gp), a plasma membrane efflux pump, has been found recently in human cultured cells [76]. However, the development of hepatotoxicity caused by drug-drug interaction is a rare side effect. In general, hepatotoxicity and liver-related disease in HIV patients are increasingly prominent in patients coinfected with hepatitis B virus (HBV), hepatitis C virus (HCV), and non-alcoholic fatty liver disease (NAFLD). Nowadays, 5–25% of patients may be coinfected with HBV, 30% with HCV, and 30–40% of patients may exhibit signs of NAFLD.

Moreover, Bilirubin is often checked to adjust dosage of cancer chemotherapy agents such as docetaxel, doxorubicin, etoposide, imatinib, irinotecan, paclitaxel, sorafenib, vincristine, and vorinostat. Besides, several antiretrovirals, such as atazanavir and indinavir are associated with unconjugated hyperbilirubinemia secondary to UGT1A1 inhibition. When assessing liver function in HIV patients on these antiretroviral agents, it is useful to also assess transaminases and alkaline phosphatase. Unconjugated hyperbilirubinemia in association with these agents and in the absence of other evidence of hepatic dysfunction may be ignored in dosing chemotherapeutic agents. On the other hand, didanosine, stavudine, and zidovudine may produce hepatotoxicity associated with lactic acidosis and steatosis. Maraviroc has been noted to rarely produce a hepatotoxicity associated with allergic features. Such hepatotoxicity should not be ignored and didanosine, maraviroc, stavudine, and zidovudine should be stopped or replaced before initiating cytotoxic chemotherapy with agents that have hepatic metabolism at standard doses, but use reduced dosing based on the degree of hepatotoxicity. The NRTIs, abacavir, emtricitabine, lamivudine, and tenofovir, and the NNRTI efavirenz are the less likely to be hepatotoxic and may often be substituted. Moreover, the risk of hepatotoxicity may increase because of the prophylaxis against OIs in patients with HIV. Severity of the side effect depend on many factors, such as the history of exposure, status of the immune system, particularly as reflected by the CD4 count, the receipt of and duration of ART, and the response to ART [77]. Prophylaxis against infection during chemotherapy may include drugs that interact with ART. Examples include the mold-active triazoles voriconazole and posaconazole. Efavirenz should not be coadministered with either voriconazole or posaconazole because it decreases the triazole AUC; ritonavir should be avoided with posaconazole. We prefer to avoid efavirenz given that it may decrease the serum concentration of triazoles, particularly because this effect may last for several weeks after efavirenz is discontinued. Thus, the combination of both anti-cancer therapy, ART, and coinfection with either HBV, HCV or NAFLD, may present many drawbacks. 

## 7. Conclusions

Here, we presented some of the most common DDIs that can occurs in HIV cancer patients undergoing a concomitant ART and chemotherapy treatment. Among the possible consequences, we analysed the effect and toxicity in liver, also caused by immunotherapeutic strategies. All of these studies and considerations are relevant to understanding the importance of the choice of the better strategies to increase patients’ compliance and efficacy of the treatment.

## Figures and Tables

**Table 1:** Drug-drug interaction between ART and chemotherapeutic agents.

Antiblastic Drug	Metabolism	Tumor	Reported Interaction
**Taxanes**			
Paclitaxel	CYP2C8, CYP3A4, and CYP3A5	KS	Co-administration of paclitaxel and CYP3A4 inhibitors, such as ritonavir can increased plasma concentration of the drug.
Docetaxel	CYP3A4		Minor adverse event with the administration of paclitaxel in combination with CYP3A4 inducers such as efavirenz (NNRTIs) and tenofovir disoproxil fumarate (NRTIs).
**The vinca alkaloids**			
Vincristine, vinblastine and vinorelbine	CYP3A4	NHL HL KS Lung cancer	Concomitant administration with protease inhibitors, can increase the plasma concentration of vinblastine leading to possible hematological and neurological side effects.
**Etoposide**	CYP3A4 with a minor contribution of CYP1A2A and CYP2E1 isoforms.	hematological malignancies and non-Hodgkin’s lymphoma	The inhibition of the CYP3A4 pathway may increase etoposide plasma concentration levels. Leading to an increased risk of mucositis, myelosuppression, and transaminitis.
**Corticosteroids**	CYP3A4	HL NHL	PIs and NNRTIs are modulators of the activity of the CYP450 enzyme system and therefore may interact with corticosteroids. PIs may increase the pharmacodynamic effects of corticosteroids when used concurrently. Conversely, CYP3A4 inducers may reduce the efficacy of these drugs.
**Alkylating Agents**			
Cyclophosphamide	CYP3A4 and CYP2B6	HD and NHL	Induction of CYP3A4 may increase neurotoxicity byincreasing the substrate availability for N-dechloroethylation.
Ifosfamide	CYP3A4 and CYP2B6		CYP3A4 inhibition could compromise its antitumor activity. CYP3A4 induction can increase the presence of toxic metabolites.
**Platin-derivates**			
Cisplatin	Primary renal elimination post Glutathione additions (GSTP1, GSTM1, and others)	Cervical cancer Lung cancer	It is not known if the combination with PIs could have an impact on toxicity and possible adverse events.
**Anthracyclines**			
Doxorubicin	Aldoketoreductase and NADPH-dependent cytochrome reductase.	HL KS NHL	Interaction between ART via CYP system appear to be unlikely.
Daunorubicin	Involved in free radical generation. Substrate of P-gp which may influence Intracellular concentrations.		
